# Evaluation of antimicrobial resistance and risk factors for recovery of intrauterine *Escherichia coli* from cows with metritis on California commercial dairy farms

**DOI:** 10.1038/s41598-022-18347-w

**Published:** 2022-08-17

**Authors:** Carl Basbas, Adriana Garzon, Noelia Silva-del-Rio, Barbara A. Byrne, Betsy Karle, Sharif S. Aly, John D. Champagne, Deniece R. Williams, Fabio S. Lima, Vinicius S. Machado, Richard V. Pereira

**Affiliations:** 1 Department of Population Health and Reproduction, School of Veterinary Medicine, University of California, Davis, Davis, CA USA; 2 Department of Pathology, Microbiology and Immunology, School of Veterinary Medicine, University of California, Davis, USA; 3 Cooperative Extension, Division of Agriculture and Natural Resources, University of California, Orland, CA USA; 4 Veterinary Medicine Teaching and Research Center, School of Veterinary Medicine, University of California, Davis, Tulare, CA USA; 5 Department of Veterinary Sciences, College of Agricultural Sciences & Natural Resources, Texas Tech University, Lubbock, TX USA

**Keywords:** Microbiology, Zoology, Diseases, Risk factors

## Abstract

The goals of this study were to evaluate factors affecting recovery and antimicrobial resistance (AMR) in intrauterine *E. coli* in post-partum dairy cows with and without metritis from commercial California dairy farms. Using a cross-sectional study design, a total of 307 cows were sampled from 25 farms throughout California, from which a total of 162 intrauterine *E. coli* isolates were recovered. During farm visits, cows within 21 days post-partum were categorized in one of three clinical presentation groups before enrollment: metritis (MET, n = 86), defined as a cow with watery, red or brown colored, and fetid vaginal discharge; cows with purulent discharge (PUS, n = 106), defined as a non-fetid purulent or mucopurulent vaginal discharge; and control cows, (CTL, n = 115) defined as cows with either no vaginal discharge or a clear, non-purulent mucus vaginal discharge. Cows diagnosed as MET had significantly higher odds for recovery of *E. coli* compared to cows diagnosed as CTL (OR = 2.16, 95% CI: 1.17–3.96), with no significant difference observed between PUS and CTL, and PUS and MET. An increase in days in milk (DIM) at the time of sampling was significantly associated with a decrease in the odds ratio for *E. coli* recovery from intrauterine swabs (OR = 0.94, 95% CI: 0.89–0.98). All intrauterine *E. coli* were resistant to ampicillin (AMP), with an AMR prevalence of 30.2% and 33.9% observed for chlortetracycline and oxytetracycline, respectively. Only 8.6% of isolates were resistant to ceftiofur (CEFT), one of the most common drugs used to treat cows on farms sampled. No significant difference in the prevalence of AMR was observed among clinical groups at the individual cow level. At the farm level, a significantly higher odds for isolating intrauterine *E. coli* resistant to chlortetracycline (OR: 2.6; 95% CI: 3.7–58.0) or oxytetracycline (OR: 1.9; 95% CI: 1.4–33.8) was observed at farms that used an intrauterine infusion of oxytetracycline as a treatment for metritis when compared to those farms that did not use this practice. Findings from this study indicate the need for further research supporting a broader understanding of farm practices driving AMR in cows with metritis, as well as data to increase the accuracy of breakpoints for AMR classification of intrauterine *E. coli* from cattle.

## Introduction

Metritis is a major uterine disease in dairy cattle, typically occurring within 21 days post-partum, characterized by an enlarged uterus, fever, and fetid, watery red-brown uterine discharge^1^. In North America, metritis impacts 10 to 30% of post-partum dairy cows^2,3^. Within the U.S., metritis is the fourth most common health issue in cows, as identified by producers^4^. Metritis has a complex etiology with various bacteria including *Escherichia coli*, *Trueperella pyogenes*, *Fusobacterium necrophorum*, and *Bacteroides* spp. associated with post-partum uterine infections^5^. Metritis negatively impacts milk production, reproductive performance, and increases the risk of culling^6^. The economic impacts of these production issues cost producers a mean of $511 per case of metritis^7^.

The most common systemic antimicrobial treatment for metritis in California is ceftiofur (CEFT), a third-generation cephalosporin with broad-spectrum activity^8^. Ceftiofur is the only antimicrobial approved by the US Food and Drug Administration (U.S. FDA) for the treatment of metritis that does not require milk to be discarded during treatment^9^. The second and third most popular antimicrobials used to treat metritis in California are ampicillin (AMP) and penicillin, respectively^8^. A survey of Midwestern dairy farms also identified CEFT as the preferred treatment for metritis, followed by AMP^10^.

Research evaluating minimum inhibitory concentrations (MIC) of *E. coli* from bovine uteri has been conducted in New York, New Zealand, and Germany using samples collected from one to seven commercial dairy farms^11–13^. While there is some research on metritis treatment preferences and diagnostic practices in California, information on MICs of intrauterine *E. coli* to common antimicrobial drugs (AMDs) used to treat metritis is lacking^8^*.* To address this knowledge gap, the goals of this study were to evaluate post-partum dairy cattle with and without metritis from commercial dairy farms in California for animal level factors affecting the recovery of intrauterine *E. coli* and evaluate and identify the animal and farm-level factors affecting the prevalence of antimicrobial resistance (AMR) in intrauterine *E. coli*. Our study hypotheses were that: (1) dairy cows diagnosed with metritis (watery, reddish or brownish, and fetid vaginal discharge) will have a significantly higher risk for isolation of intrauterine *E. coli* when compared to cows with non-fetid purulent or mucopurulent vaginal discharge (PUS), or cows with clear lochia, clear mucus, or no vaginal discharge (CTL); (2) dairy cows diagnosed with metritis will have a significantly higher risk for isolation of AMR intrauterine *E. coli* when compared to PUS or CTL cows; (3) farm management practices related to diagnosis and treatment of metritis will be significantly associated with farm-level prevalence of AMR in intrauterine *E. coli*. This is the first study to report MIC data for intrauterine *E. coli* from post-partum dairy cows with metritis housed on multiple (n = 25) commercial dairy farms in California. Additionally, this is one of the first studies of AMR prevalence within intrauterine *E. coli* recovered from post-partum dairy cows to use the most recently updated Veterinary Clinical & Laboratory Standards Institute (CLSI) MIC breakpoints^14^ and CLSI guidelines related to MIC breakpoints for veterinary pathogens^15^.

## Results

### Descriptive data

The number of *E. coli* samples recovered and information on animal samples by farm is presented in Table 1. A total of 307 cows were sampled from the 25 enrolled farms. All enrolled farms had at least one cow assigned to each of the three clinical classifications of vaginal discharge, except for two farms where no MET cows were identified during our visit. DIM at time of diagnosis for cows with culture positive results for *E. coli* for MET, PUS, and CTL were 8.1 (95% CI 7.1–9.1), 10.6 (95% CI 9.1–12.0), and 10.6 (95% CI 8.9–12.3), respectively.

**Table 1:** Distribution of *E. coli* (n = 162) isolated from intrauterine swabs collected at 25 commercial dairy farms by clinical presentation group (CTL, MET, and PUS).

Farm	CTL^a^ %, (A/B)^b^	MET^a^ %, (A/B)^b^	PUS^a^ %, (A/B)^b^	TOTAL %, (A/C)^c^
1	20 (1/5)	0 (0/2)	0 (0/4)	9 (1/11)
2	75 (3/4)	75 (3/4)	0 (0/3)	55 (6/11)
3	50 (2/4)	100 (5/5)	67 (2/3)	75 (9/12)
4	0 (0/3)	0 (0/0)	33 (2/6)	22 (2/9)
5	60 (3/5)	50 (1/2)	20 (1/5)	42 (5/12)
6	40 (2/5)	0 (0/0)	100 (1/1)	50 (3/6)
7	75 (3/4)	67 (2/3)	60 (3/5)	67 (8/12)
8	20 (1/5)	50 (1/2)	0 (0/3)	20 (2/10)
9	50 (2/4)	33 (1/3)	50 (2/4)	45 (5/11)
10	60 (3/5)	50 (1/2)	40 (2/5)	50 (6/12)
11	0 (0/5)	40 (2/5)	40 (2/5)	27 (4/15)
12	20 (1/5)	60 (3/5)	0 (0/5)	27 (4/15)
13	40 (2/5)	60 (3/5)	60 (3/5)	53 (7/15)
14	40 (2/5)	20 (1/5)	40 (2/5)	33 (5/15)
15	20 (1/5)	100 (4/4)	40 (2/5)	50 (7/14)
16	25 (1/4)	75 (3/4)	33 (2/6)	43 (6/14)
17	100 (5/5)	75 (3/4)	100 (4/4)	92 (12/13)
18	100 (2/2)	100 (3/3)	50 (2/4)	78 (7/9)
19	40 (2/5)	50 (1/2)	80 (4/5)	58 (7/12)
20	60 (3/5)	60 (3/5)	40 (2/5)	53 (8/15)
21	80 (4/5)	60 (3/5)	75 (3/4)	71 (10/14)
22	60 (3/5)	100 (5/5)	100 (5/5)	87 (13/15)
23	80 (4/5)	100 (5/5)	60 (3/5)	80 (12/15)
24	20 (1/5)	80 (4/5)	100 (1/1)	55 (6/11)
25	60 (3/5)	100 (1/1)	67 (2/3)	67 (6/9)
TOTAL	47 (54/115)^d^	67 (58/86)^d^	47 (50/106)^d^	53 (162/307)

A, swabs positive for *E. coli*; B, total number of swabs collected from cows in clinical group; C, total number of swabs collected at each farm.

^a^Clinical presentation of cows when intrauterine samples were collected.

^b^Percentage, (Swabs positive for *E. coli* / total number of swabs collected from cows in clinical presentation group).

^c^Percentage, (Swabs positive for *E. coli* / total number of swabs collected at each farm).

^d^Percentage, (Swabs positive for *E. coli* / total number of swabs from all farms for cows in clinical group).

### Risk factors for E. coli isolation

Risk factors for *E. coli* isolation are presented in Table 2 and supplemental table 1. The odds ratio for isolating intrauterine *E. coli* isolate from MET cows when compared to CTL cows was 2.0 (95% CI: 1.1–3.7, *P* value = 0.03). No significant difference between cows diagnosed as PUS and CTL, or a MET and PUS was observed for isolation of *E.coli* from intrauterine swabs. Days in milk of the cow sampled were significantly associated with lower odds of isolating intrauterine *E. coli* for each day increase in DIM for MET and PUS cows (Table 2).

**Table 2:** Summary of the logistic regression model evaluating the effect of the clinical presentation groups (MET, PUS, or CTL) and the days in milk (DIM) on the odds ratio of isolation of *E. coli* from intrauterine swabs collected from cows at 25 commercial dairy farms.

Variable	Odds ratio	OR (95% Confidence interval)		*P* value
		Lower	Upper	
**Clinical group**^**a**^				0.005
MET vs PUS	1.67	0.87	3.2	0.11
MET vs CTL	2.00	1.07	3.7	0.03
PUS vs CTL	1.19	0.68	2.1	0.53
DIM^2^				0.0008
**Clinical group**^**a**^ * DIM^**b**^				0.02
MET	0.85	0.71	0.98	0.01
PUS	0.88	0.80	0.96	0.004
CTL	0.99	0.93	1.06	0.92

^a^Clinical presentation group (MET, PUS, or CTL) of cows when intrauterine samples were collected. (MET) metritis discharge defined as a watery, red or brown colored, and fetid vaginal discharge; (PUS) purulent discharge defined as a non-fetid purulent or mucopurulent vaginal discharge; and (CTL) control, healthy discharge defined as cows with either no vaginal discharge, clear mucus, or clear lochia.

^b^Days in Milk at sampling time.

### E. coli antimicrobial resistance

The distribution of minimum inhibitory concentration (MIC) and resistance for intrauterine *E. coli* (n = 162) by individual drug for the BOPO6F panel are shown in table 3. The most common resistance profiles and the resistance profile for each isolate and are presented in supplemental tables 2 and 3, respectively. No significant association (*P* > 0.05) was observed at the animal level between *E. coli* AMR for the nine drugs with clinical breakpoints and the clinical presentation group.

**Table 3:**
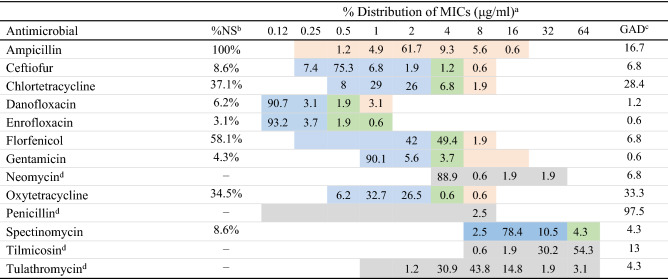
Distribution of minimum inhibitory concentration (MIC) and resistance for intrauterine *E. coli* (n = 162) by individual drug for the BOPO6F panel.

Highlighted areas in blue corresponds to susceptible, green corresponds to intermediate, and orange corresponds to resistant classification. For antimicrobials without a MIC breakpoint, the dilution scale tested is highlighted in gray. For the lowest MIC value in the dilution range, results indicate lowest MIC detected, but should be interpreted as less than or equal to ( ≤) the lowest MIC detected.

^a^Distribution of minimum inhibitory concentration (MIC).

^b^Percent of isolates classified as non-susceptible (Intermediate and Resistant) to the referred antimicrobial drug (%NS).

^c^Percent of bacterial growth in all antimicrobial dilutions tested (GAD), Read as MIC > highest drug concentration available.

^d^*Enterobacterales* are highly susceptible to these drugs or no CLSI breakpoint available.

The percent of all *E. coli* isolates classified as susceptible for the four antimicrobial drugs commonly used to treat cows with metritis in the U.S.^8^, are presented in Fig. 3. Of the nine antimicrobials tested with available MIC breakpoints, AMP had the highest prevalence of AMR, with all isolates being classified as resistant (Table 3). Although all isolates were resistant to ampicillin, nearly 60% of isolates (n = 97) were resistant to AMP alone (supplemental table 10. A total of 8.6% of isolates (n = 12) included ceftiofur resistance within their total AMR resistance profile (supplemental table 2).

The second most common resistance profile was ampicillin-chlortetracycline-oxytetracycline (18.5% of isolates). A total of 3.1% of isolates (n = 5) displayed resistance to ampicillin-ceftiofur-chlortetracycline-florfenicol-oxytetracycline and 2.5% of isolates (n = 4) displayed resistance to ampicillin-ceftiofur-chlortetracycline-oxytetracycline (supplemental table 2).

**Figure 1:**
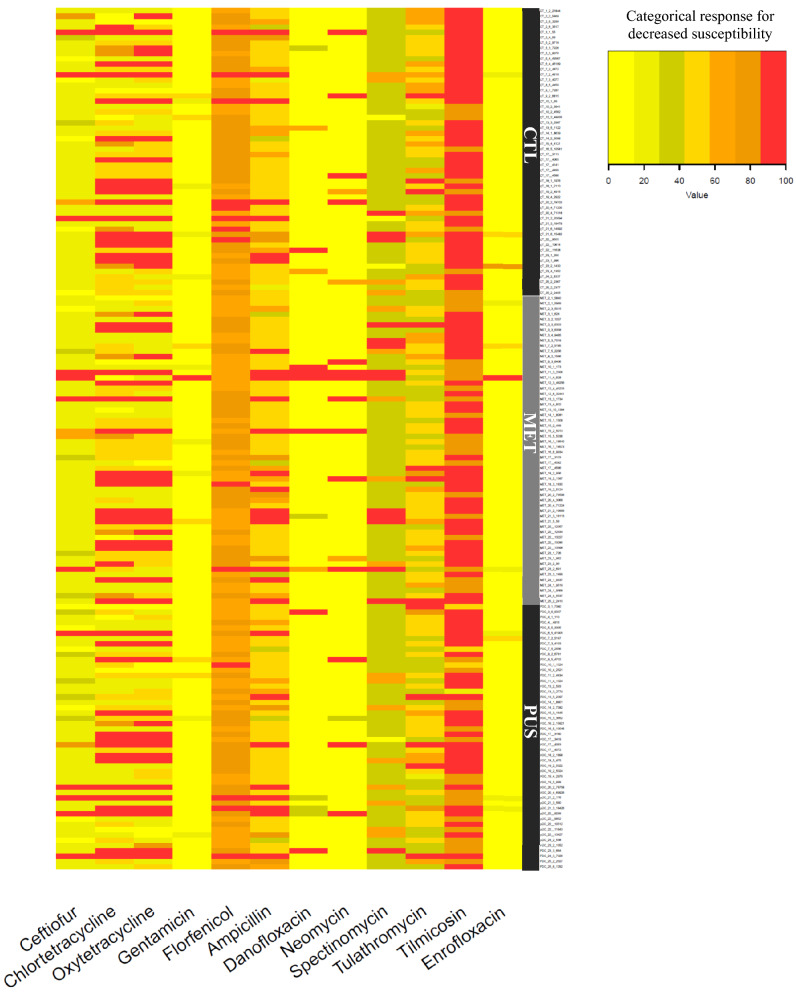
Heat map of MICs for 12 antimicrobials compared against 162 *E. coli* isolates grouped by clinical presentation group (CTL, MET, PUS). Each row represents an isolate that was categorized by percent decrease in the susceptibility range.

**Figure 2:**
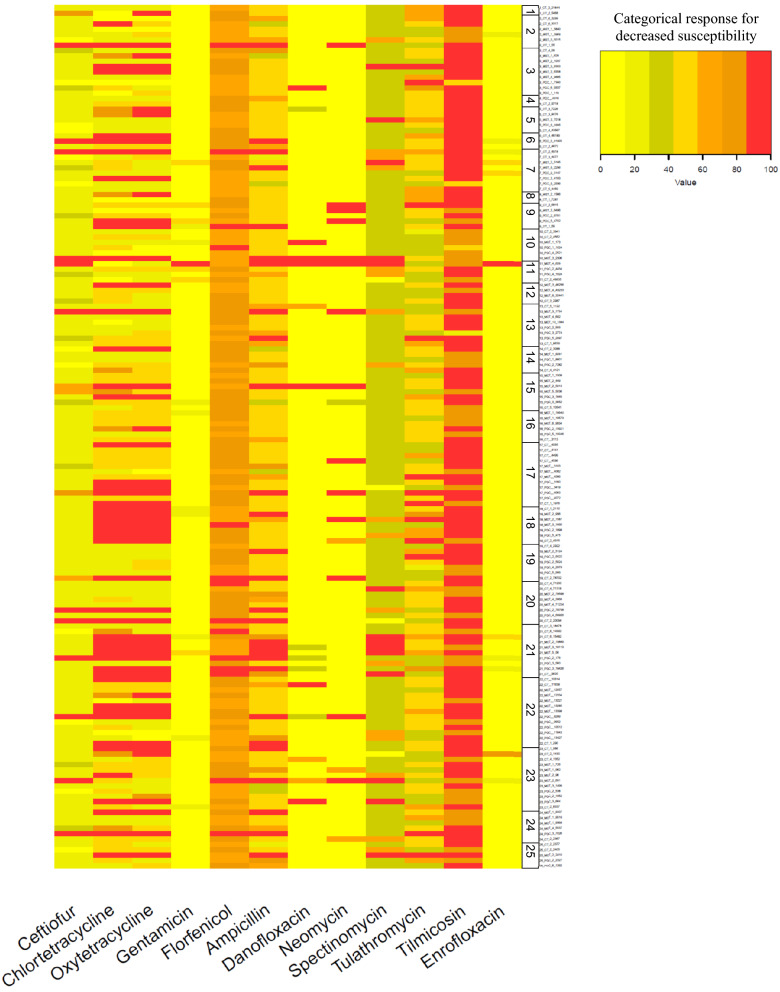
Heat map of MICs for 12 antimicrobials compared against 162 *E. coli* isolates grouped by the farm (n = 25). Each row represents an isolate that was categorized by percent decrease in susceptibility range. Boxed numbers indicate which of the 25 farms samples correspond.

Heat map of MICs for antimicrobials for the 162 *E. coli* isolates grouped by clinical presentation group (CTL, MET, PUS) and farm, are shown in Figs. 1 and 2, respectively. When visually comparing CTL, MET, and PUS groups in Fig. 1, no clinical presentation group had a noticeable visual trend for percentile distribution toward higher MIC quantiles. When comparing farms in Fig. 2, there was a visual clustering of isolates in a higher MIC quantile for specific farms, particularly for chlortetracycline and oxytetracycline for farms 18 and 21, which were the only farms that used tetracycline drugs as their primary treatment for metritis in dairy cows (Table 4).

**Table 4:**
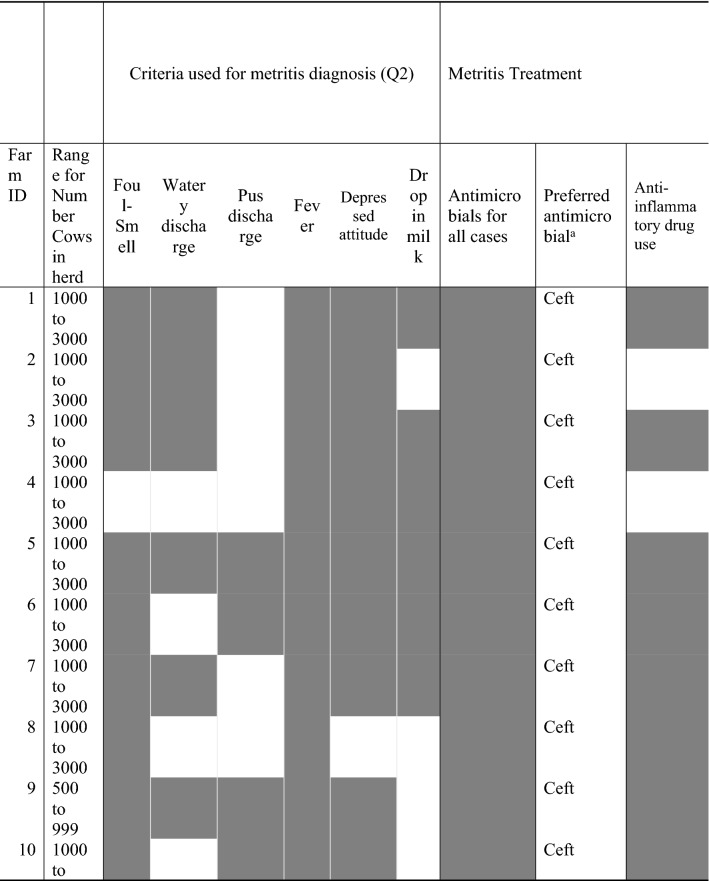

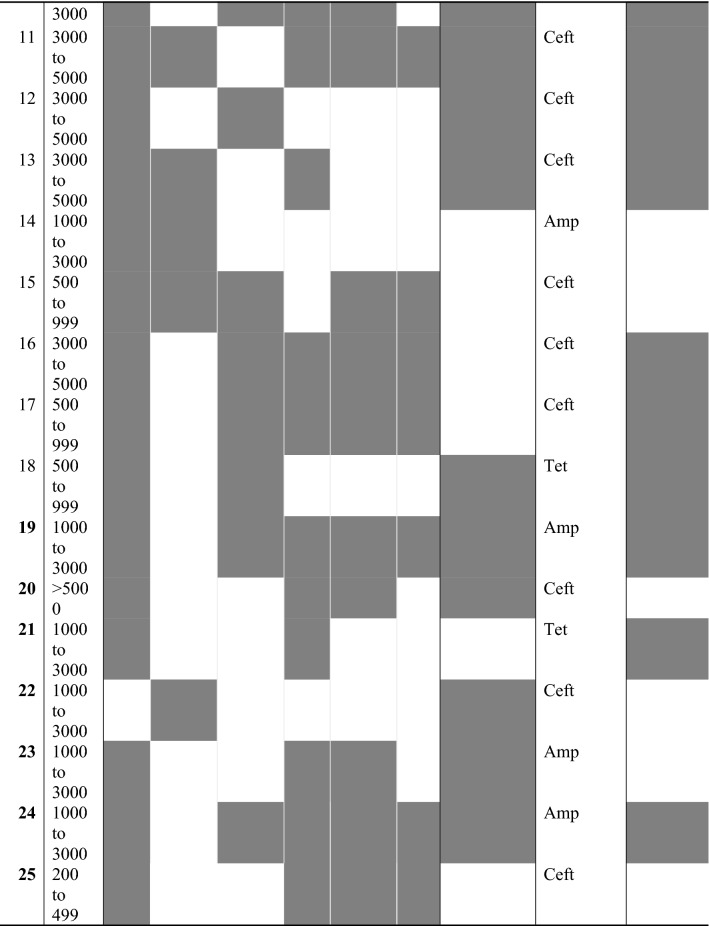
Farm level factors related to the number of dairy cows and metritis diagnosis and treatment criteria.

Grey filled cells indicate a yes to the questions.

^a^Ceft, ceftiofur; Amp, ampicillin; Tet, tetracycline.

### Antimicrobial treatment on E. coli AMR

A total of 11% (18/162) of animals sampled for which *E. coli* was isolated received antimicrobial treatment within the fourteen days prior to sampling. For cows diagnosed with metritis, 26% (12/46) had a prior treatment with antimicrobials, and 11% (5/45) and 2% (1/53) for PUS and CTL cows, respectively. Based on survey data collected for farms, we would expect a higher number of cows with metritis being treated; however, our research team selected cows for enrollment independent of prior diagnosis of metritis, resulting in a metritis diagnosis occurring prior to that of the farm. This is not surprising, given that most farms used systemic signs of disease to diagnose metritis (e.g., depressed attitude, drop in milk) (Table 4). Indicating a more severe case; the approach used by our study was based on vaginal discharge, which allows an earlier diagnosis of metritis, and therefore explaining the reason for metritis cases identified in not having received a prior antimicrobial treatment by the farm.

Results for Fisher’s Exact Test analysis evaluating the effect of individual animal treatment with antimicrobials is shown in supplemental table 4, and was not associated with a significant increase of AMR in *E. coli*. When this analysis was stratified by clinical presentation, again, no significant effect of individual animal treatment on AMR within *E. coli* was observed. Results for Fisher’s Exact Test analysis evaluating the effect of individual animal treatment with ceftiofur alone is shown in supplemental table 5; Individual animal treatment with only ceftiofur 14 days prior to sampling did not result in any significantly increased odds ratios for antimicrobial resistance to the nine antimicrobials analyzed. When this analysis was conducted stratifying by clinical presentation group (CTL, MET, and PUS), there were also no significantly increased odds for antimicrobial resistance to the seven antimicrobials analyzed for (supplemental table 6).

**Table 5:** Association of farm-level management of using oxytetracycline as an intrauterine infusion as the most common drug for the treatment of metritis and farm-level prevalence of AMR to tetracycline drugs in intrauterine *E. coli.*

Farm-level risk factors	Chlortetracycline				Oxytetracycline			
	Estimate^a^	LSM^b^	*SEM*^c^	*P value*^4^	Estimate^a^	LSM^b^	*SEM*^c^	*P value*^4^
**Intrauterine treatment with oxytetracycline**^**4**^				0.0005				0.019
No	Ref	0.82	0.09		Ref	0.70	0.14	
Yes	2.68	0.03	0.03		1.94	0.25	0.04	

^a^Parameter estimate for the multivariate model evaluating resistance to the referred drug.

^b^Least-square means (LSM) of farm-level prevalence of intrauterine *E. coli* resistant to the referred drug.

^c^Standard error of the means (SEM) for the LSM.

^4^*P* value from analysis comparing farm-level prevalence of AMR between farms for the referred drug, adjusted using Bonferroni.

Results for the Fisher’s Exact Test evaluating the effect of individual animal treatment with tetracycline alone is shown in supplemental table 7; individual animal treatment with tetracycline 14 days prior to sampling did not result in any significantly increased odds ratios for antimicrobial resistance to the nine antimicrobials analyzed. When this analysis was conducted stratifying by clinical presentation group (CTL, MET, and PUS), there were also no significantly increased odds for antimicrobial resistance to the seven antimicrobials analyzed for (supplemental table 8).

The mixed-effect multinomial logistic regression model used to evaluate the association between intrauterine *E. coli* AMR and animal-level variables, did not identify treatment group or any other animal-level variable evaluated, including prior antimicrobial treatment, as being significantly associated with AMR in *E. coli*.

### Farm-level antimicrobial treatment and management practices

Descriptive data by farm related to the number of dairy cows and metritis diagnosis and treatment criteria are presented in Table 4. Farms in which cows were treated for metritis using intrauterine treatment with oxytetracycline had a significantly higher farm-level prevalence of intrauterine *E. coli* with AMR to oxytetracycline (LSM ± SEM: 0.82 ± 0.09) and chlortetracycline (LSM ± SEM: 0.70 ± 0.14) when compared to farms in which cows were not treated for metritis using intrauterine treatment with oxytetracycline (LSM ± SEM: 0.03 ± 0.03 and LSM ± SEM: 0.25 ± 0.04, respectively) (Table 5).

## Discussion

### Recovery of E. coli from intrauterine swab samples

*E. coli* was recovered from 53% of intrauterine swab samples collected from all post-partum dairy cows in our study, which is within the range previously observed by other researchers. Bicalho et al.^16^, recovered 125 (33.4%) intrauterine *E. coli* from 374 total lactating Holstein cows sampled in upstate New York [a subset of 117 cows displayed clinical signs of metritis]. In another study, De Boer et al.^12^, recovered 209 (76.8%) intrauterine *E. coli* from 272 pasture-raised cows in New Zealand; and Kasse et al.^17^, recovered 156 (42%) intrauterine *E. coli* from 371 Holstein dairy cows in Canada. Furthermore, recovery of *E. coli* was higher in cows with MET when compared to PUS and CTL.

MET cows had significantly higher odds ratio for isolation of intrauterine *E. coli* when compared to CTL cows (Table 2), and is in agreement with previous studies^17,18^. A study by Pohl et al. (2018) that isolated intrauterine *E. coli* from cows using two clinical signs to define metritis (reddish-brown fetid discharge and rectal temperature > 39.5 °C) had a 90% recovery of intrauterine *E. coli*, and 70% recovery when using solely one clinical sign (reddish-brown fetid discharge or rectal temperature > 39.5 °C). In contrast, cows not displaying clinical signs of metritis had an *E. coli* recovery of 54%^13^. The Pohl et al.^13^ study also observed that cows with two clinical signs of metritis had 7.16 times the odds of having intrauterine *E. coli* compared to cows without metritis, in agreement with our findings. The difference in the magnitude of *E. coli* recovery in cows with metritis between Pohl et al.^13^ and our study may be explained by differences in herd management practices and geographical factors from German dairy farms that resulted in a different magnitude of recovery of *E. coli* in cows with metritis.

A higher days in milk (DIM) at the time of MET or PUS diagnosis was found to be significantly associated with a lower odds of isolation of intrauterine *E. coli* (Table 2). The relationship between DIM and odds to isolate *E. coli* agrees with previous findings^19^, where the progression of the uterine microbiota from calving was evaluated, with an observed rapid decrease in the relative abundance of Proteobacteria, a major phylum of Gram-negative bacteria that includes *E. coli*, from 0 to 6 ± 2 DIM. Jeon et al.^19^ also observed a subsequent increase in the relative abundance of bacteria in the phylum Bacteroidetes from 0 to 6 ± 2 DIM. While the dynamics of the uterine microbiome are complicated, particularly at the time of parturition, increases in relative abundance of other microbes likely drive the decrease in abundance of Proteobacteria; therefore decreasing the odds of isolating intrauterine *E. coli*^20^. A definitive explanation behind this phenomenon remains elusive and continues to be a topic of research.

### *E. coli* AMR

When comparing CTL, PUS, and MET cows, no significant difference in the odds ratio for isolating AMR *E. coli* isolates to the AMDs tested was observed. This is in discordance with our hypothesis, that had an assumption that cows with MET may have been colonized by *E. coli* carrying both virulence and antimicrobial resistance genes. Previous studies have shown that specific virulence genes are associated with intrauterine *E coli* isolated from cows with metritis^16^. Furthermore, a study from cows from a single farm, using whole genome sequencing to characterize intrauterine *E. coli*, observed a correlation between intrauterine pathogenic *E. coli* (characterized based on presence of virulence genes) and extended spectrum β-lactamase (ESBL) genes, which confer resistance to expanded-spectrum cephalosporins^21^. Discordance between our results using phenotype methods for AMR diagnosis and those using genomic approaches could be due to disagreements that have been reported between these two methods.

Resistance to ceftiofur, the most common systemic antimicrobial treatment for metritis in California, was low in our study with 8.6% of isolates (n = 12) phenotypically resistant^8^. Similar studies conducted in New York State, New Zealand, and Germany also observed low AMR (as specified by CLSI breakpoints available at the time of publication) to CEFT within uterine *E. coli*; with 1.2%, 0%, and 5.9% of isolates resistant, respectively^11–13^.

Extra-label use of ampicillin has previously been reported as the second most common treatment option for metritis in California dairy cows^8^. In the literature, intrauterine susceptibility of *E. coli* to AMP has varied, possibly due in part to the use of breakpoint values that have been periodically updated^12^. As an example, a study by^11^ observed that approximately 34% of early post-partum cows harbored ampicillin-resistant *E. coli*. Due to the lack of specific MIC breakpoints for *Enterobacterales* from cattle for AMP at the time, this study used a resistance breakpoint of ≥ 16 μg/mL based on a CLSI breakpoint used for human isolates^22^. The use of human-based CLSI breakpoints has been a common standard in veterinary studies evaluating MIC, with specific breakpoints against AMP for *E. coli* from cattle only being available starting in 2018 with the release of the 4th edition of “Performance Standards for Antimicrobial Disk and Dilution Susceptibility Tests for Bacteria Isolated From Animals” allowing for SIR classification of *Enterobacterales* for ampicillin^23^. As an example, had we used the human clinical breakpoint for ampicillin for *Enterobacterales*, as per the CLSI M100 31st edition for which the breakpoint for resistance is MIC ≥ 32 µg/mL, only 16.7% of our isolates would have been classified as resistant. This large discrepancy is a reflection of how MIC breakpoints are defined for *Enterobacterales* in humans and animals. Traditionally, MIC breakpoints are set using a range of data, including in vitro microbiological data, animal and human pharmacokinetic/pharmacodynamic (PK/PD) data, and clinical and bacteriological outcome data from prospective clinical studies^24^. Furthermore, the accuracy of the data used to determine MIC breakpoints for specific animal species and tissues will directly affect the validity of the results related to SIR classification.

Oxytetracycline is approved in the US for the systemic treatment of metritis caused by species of staphylococci and streptococci. A study by^12^ reported that of 209 intrauterine *E. coli,* 83.2% of were susceptible and 4.8% were resistant to oxytetracycline. In Germany, a study reported that of 85 intrauterine *E. coli* isolates*,* 81.1% were susceptible and 9.5% were resistant to tetracycline^13^. In our study, 30.3% of *E. coli* tested against chlortetracycline and 33.9% of *E. coli* tested against oxytetracycline were classified as resistant. The observed higher prevalence of resistance to tetracyclines in our study when compared to previous studies may reflect specific practices for managing metritis in California. In Fig. 2, the visual clustering of isolates in a higher MIC quantile collected from the two farms that indicated using tetracycline drugs as their first choice for treatment of metritis in dairy cows suggests that treatment of cows with metritis using tetracyclines may increase the selection of AMR to drugs in that class. Future studies should be designed to allow for the determination of causation.

Our study revealed that using oxytetracycline intrauterine infusion as a treatment tended to increase the odds of recovering intrauterine *E. coli* carrying resistance to either chlortetracycline or oxytetracycline. In California, approximately 27% of farms used intrauterine infusion with oxytetracycline for treatment of metritis^8^. However, researchers have recommended against intrauterine infusions due to lack of evidence to support any added benefit in reproduction or cure^25,26^. Given the extra-label nature of the use of intrauterine oxytetracycline in cattle, this practice can only occur though the prescription of a veterinarian, and appropriate milk withhold periods should be followed^27^.

**Figure 3:**
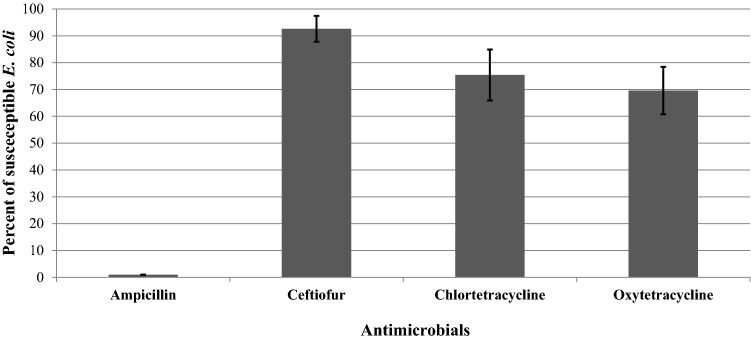
Percent of *E. coli* isolates susceptible to commonly used antimicrobial treatments for metritis. Current Clinical Laboratory Standard Institute veterinary breakpoints were used to define susceptibility^14^. A total of 162 *E. coli* isolates were obtained from the uterus of post-partum cows housed in 25 California dairies.

The fluoroquinolone enrofloxacin is approved for use in treating respiratory disease in non-lactating dairy cattle under 20 months of age, however, its extra-label use to treat bovine metritis is illegal^28^. Due to drug dilutions present on the plate, we could only classify isolates as susceptible and intermediate. As such, we can only report that enrofloxacin susceptibility was high with 96.9% of isolates classified as susceptible (Fig. 3). None of the farms in our study reported using enrofloxacin as a treatment option for metritis. Our findings suggest very low resistance of intrauterine *E. coli* to enrofloxacin, supporting the expected lack of use of this drug in lactating dairy cattle. Fluoroquinolone resistance within our isolates could potentially be originating from horizontal gene transfer or from co-selection due in part to the use of approved AMDs to treat metritis; however, further study to investigate this would be necessary^29,30^.

While resistance to each of the antimicrobials discussed above is concerning, multidrug resistance (MDR) is an especially pressing issue if an isolate is resistant to multiple common treatment options. The most common resistance profile was to AMP alone (59.9% of isolates), while resistance to ampicillin-chlortetracycline-oxytetracycline (18.5% of isolates) was the 2nd most common profile. Our study showed the presence of resistance to the common drugs used to treat metritis in California with 2.5% of isolates (n = 4) resistant to ampicillin-ceftiofur-chlortetracycline-oxytetracycline. The relatively low prevalence of MDR within intrauterine *E. coli* isolates contrasts with Santos et al. (2010) in which a total of 35% (n = 80) of *E. coli* isolates from cows with metritis were MDR, with the major MDR profile being ampicillin-chloramphenicol-florfenicol resistance, observed in 96.4% of MDR isolates^11^. However, our results do more closely resemble those of Abdelfattah et al.^31^ in which a total of 14.14% (n = 307) of 2171 *E. coli* isolates, recovered from fecal samples from healthy, adult dairy cows from 10 farms in California, were MDR. Their major MDR profile being florfenicol-sulphadimethoxine (16.2%), and tetracycline-florfenicol-sulphadimethoxine (6.82%).

For farm level analyses, an important consideration when extrapolating results, is that the results represent findings for a specific population within the herd. Specifically, fresh cows (within the first 21 days in milk) that were sampled and also had an *E. coli* culture-positive result (cows without a culture positive result were not included in this analysis). Within these constraints, our results aimed to have internal validity for this specific population.

## Conclusion

An increase in DIM at the time of sampling was significantly associated with a decreased odds for *E. coli* recovery, while classification within the MET clinical presentation group at the time of sampling was significantly associated with increased odds of recovery of *E. coli* from intrauterine swabs. A low prevalence of AMR to CEFT, the most common metritis treatment in our study, within *E. coli* was observed. The extra-label use of intrauterine infusion with oxytetracycline on two farms was observed as a significant factor for increased farm-level prevalence of intrauterine *E. coli* with AMR to oxytetracycline and chlortetracycline, highlighting the potential impacts of this practice on antimicrobial stewardship. Our findings support the need for further research to support a better understanding of farm practices driving AMR in cows with metritis, as well as data to support breakpoints that will result in more accurate AMR identification within intrauterine *E. coli* from cattle.

## Materials and methods

The University of California Institutional Animal Care and Use Committee (IACUC; #20620) approved all experimental procedures conducted with animals for this study. The UC Davis Institutional Review Board (IRB) Administration granted an exemption (IRB ID 1307716-1) for all experimental procedures for this study.

### Study design

A convenience sample of 25 commercial dairy farms from the Sacramento and San Joaquin Valleys in California was recruited with the help of local veterinarians and UC Davis faculty and extension advisors. The study was conducted between September 2018 and November 2019.

Using a cross-sectional study design, intrauterine swabs were collected from post-partum cows between 3 and 21 DIM; cows that were unable to stand were not eligible for enrollment in the study. Three clinical presentation groups were defined based on vaginal discharge (VD) characteristics^32^ as metritis discharge (MET): watery, reddish or brownish, and fetid), purulent discharge (PUS): non-fetid purulent or mucopurulent vaginal discharge), and normal discharge (CTL): clear lochia, clear mucus, or no vaginal discharge. Due to sampling time limitations, five cows per clinical group were targeted as the maximum number per dairy.

Researchers visited each of the 25 farms once during the morning lockups of fresh cow pens, while farm employees were performing their own health checks. Researchers (R.V.P. and A.G.) collected vaginal discharge from cows using a Metricheck™ device (Simcrotech, Hamilton, New Zealand) cleaned with 2% chlorhexidine gluconate solution between cows. The VD was evaluated by sight and smell, and cows were assigned to the corresponding clinical presentation group. Evaluation of animals was conducted independent of data from the farm on prior diagnosis of metritis, and were conducted independently of farm employee findings.

For animals selected to be enrolled in the study, rectal temperature was measured using the GLA M900 thermometer (GLA Agricultural Electronics, San Luis Obispo, CA, USA). Prior to intrauterine sample collection, researchers cleaned the vulva using dry paper towels and 70% isopropyl alcohol. A 30-inch double-guarded sterile culture swab (McCullough; Jorgensen Labs Inc., Loveland, CO, USA) was gently passed through the vulva and cervix until reaching the uterine body. The swab was exposed and rolled against the uterine wall, retracted within the double sheath, removed from the cow, and immediately placed in Amies transport media with charcoal (BBL™ CultureSwab™ Plus; Becton, Dickinson, and Company, Sparks, MD, USA). The swabs were kept on ice until inoculation on to solid medium in the laboratory within 24 h of sample collection. Individual animal antimicrobial treatment in the last 14 days for animals sampled was recorded.

A sample size calculation was conducted based on our first hypothesis that dairy cows diagnosed with metritis will have a higher risk for isolation of intrauterine *E. coli* when compared to CTL cows. For this purpose, an a priori sample size estimation for the proportion of *E. coli* culture positive cows in MET and CTL was made based at a 90% power (σ: 0.1; α: 0.05; μ_MET._: 0.7, μ_CTL_: 0.4) was calculated in JMP Pro 16.0 (SAS Institute Inc., Cary, NC), resulting in a minimum of 53 animals per clinical group.

### Survey and treatment records

A survey questionnaire was developed to collect information on farm characteristics, management practices, and antimicrobial treatment regarding metritis. The survey was administered by R.V.P or A.G. and targeted dairy managers and animal handlers. On-farm antimicrobial treatment history for each sampled cow for the last fourteen days was collected via either electronic records or after interviewing workers during farm visits. Data collected from interviews were entered into spreadsheets for analysis (Microsoft Office Excel 2010, Microsoft Corp., Redmond, WA).

### Bacterial isolation and antimicrobial susceptibility testing

Within 24 h after collection, each uterine swab was used to inoculate a single CHROMagar-*E. coli* selective plate (CHROMagar Microbiology, Paris, France) which was then incubated at 37 °C for 24 h. A single, isolated colony was chosen at random and subcultured in 10 mL of brain heart infusion broth (Difco; Becton, Dickinson, and Company, Sparks, MD, USA) at 37 °C for 24 h. The broth culture (500 µL) was mixed with 50% sterile glycerol/50% sterile water solution (500 µL) prior to storage at − 80 °C.

For all isolates, antimicrobial susceptibility testing was conducted in batches after completion of sample collection using a broth microdilution method following the Clinical Laboratory Standards Institute (CLSI) guidelines^14^. The Sensititre Vet Bovine/Porcine plate (BOPO6F, Trek Diagnostic Systems, Oakwood Village, OH, USA) was used for testing susceptibility to the following antimicrobial drugs: penicillins (penicillin and ampicillin), cephalosporins (ceftiofur), fluoroquinolones (danofloxacin and enrofloxacin), phenicols (florphenicol), sulfas (sulphadimethoxine and sulfamethoxazole/trimethoprim), tetracyclines (chlortetracycline and oxytetracycline), macrolides (tylosin tartrate, tulathromycin, and tilmicosin), aminoglycosides (gentamicin and neomycin), lincosamides (clindamycin), pleuromutilins (tiamulin), and aminocyclitols (spectinomycin). Sensititre plates were read manually, and minimum inhibitory concentrations were interpreted using current CLSI breakpoints when available^14^ (supplemental table 9).

Prior to the veterinary CLSI guidelines, the only option to define the susceptible, intermediate or resistant (SIR) classification of *E. coli* isolates from cows with metritis was to utilize human-based breakpoints, which have been used for currently available studies in literature. However, as defined by CLSI VET09 guidelines for extrapolating breakpoints for veterinary pathogens, the use of human-based breakpoints result in SIR interpretations that have very low confidence, and are not recommended^15^. Instead, as per Chapter 8 of the CLSI VET09 document, which focuses on bovine-specific breakpoints**,** the recommendation for defining breakpoints when they are not available for specific bacteria or anatomical locations, is to apply a different bacterial species or infection site from a bovine-specific source. Based on these guidelines by CLSI, we utilized this updated approach to maximize the accuracy of SIR classification of isolates in the study (supplemental table 9).

### Data management and statistical analysis

CLSI MIC breakpoints were available for only nine of the 18 AMDs tested (ampicillin, ceftiofur, chlortetracycline, oxytetracycline, florfenicol, gentamicin, danofloxacin, spectinomycin, and enrofloxacin) (supplemental table 9). The nine drugs tested and later used in the analysis had CLSI breakpoints for either *Enterobacterales* or *Pasturella multocida* in cattle and horses, as recommended by CLSI. Isolates that grew in all dilutions of an antimicrobial assessed were classified as “Growth in all dilutions” (GAD) because their MIC was higher than the highest dilution tested in our study (supplemental Fig. 1). By using GAD, we stratified the data between isolates for which the highest concentration in the plate was the actual MIC (e.g., CEFT for isolates where the MIC = 8) from those that grew at the highest concentration available on the MIC plate (e.g., CEFT for isolates where the MIC > 8), for which the actual MIC value is unknown. Antimicrobial drug resistance profiles for intrauterine *E. coli* isolates are presented in supplemental table 2.

### Risk factors for* E. coli* isolation

A logistic regression model in SAS (SAS Institute Inc., Cary, NC; version 7.15) using PROC GLIMMIX logit function was used to evaluate animal-level risk factors collected at the time of sampling on the odds of isolating *E. coli* from an intrauterine swab sample. The dependent variable was the binomial variable for culture-positive or negative for *E. coli*, and the independent variables were clinical presentation group (MET, PUS, or CTL), lactation number (1, 2, and 3 or greater), days in milk (DIM), and fever (categorical variable, with 39.5 ºC as a fever benchmark) at time of sampling^1^. All interactions were considered in the model. Univariate analysis for each explanatory variable was conducted; all variables with a *P* < 0.3 were selected to be offered to the model using a backward stepwise elimination process. Farm was controlled as a random effect in the model. The quadratic association between DIM and outcomes of interest were evaluated and retained in the model if significant. Pairwise comparisons between the clinical presentation groups were conducted, adjusting for multiple comparisons using the Tukey–Kramer approach. The Akaike information criterion (AIC) was used for model selection and to ensure a more parsimonious model was selected. Clinical presentation group was forced into all models regardless of the *p*-value. A variable was considered a confounder if the coefficient of a significant variable in the model changed ≥ 20% after removal from the model. All models included farm as a random effect.

### Evaluation of antimicrobial treatment on AMR prevalence

Univariate analysis was conducted to evaluate the effect of individual animal antimicrobial treatment on AMR in *E. coli* (n = 162), independent of clinical presentation group, as well as by stratifying the analysis by treatment group. A binary variable was created for being treated in the preceding 14 days prior to sampling with any antimicrobial drug. Because most animals that had an *E. coli* isolate and received any antimicrobial treatment were treated with ceftiofur or tetracycline (17/18), antimicrobial specific binary variable were created, where animals were either treated with that specific antimicrobial or did not receive that specific antimicrobial (supplemental table 10). Fisher’s Exact Test analysis was used to evaluate the effect of individual animal treatment with any antimicrobials (supplemental table 4), only ceftiofur (supplemental table 5, supplemental table 6), or only tetracyclines (supplemental table 7, supplemental table 8) on AMR in *E. coli* for all antimicrobials tested.

### Risk factors for* E. coli* antimicrobial resistance

A logistic regression model using the Logit function in PROC GLIMMIX in SAS (SAS Institute Inc., Cary, NC) was used to evaluate the association between intrauterine *E. coli* AMR and animal-level variables. Univariate analysis between each explanatory variable and the categorical binomial variables for ampicillin, ceftiofur, chlortetracycline, oxytetracycline, florfenicol, gentamicin, danofloxacin, spectinomycin, and enrofloxacin as resistant or susceptible was used to identify tests with a *P* < 0.3; these were selected to be offered to the model using a backward stepwise elimination process. A model was generated for each of the nine AMDs with available breakpoints using a categorical binomial variable to classify an isolate as resistant or susceptible. Independent individual animal-level variables offered to the model were clinical presentation group (retained in all models), lactation number, rectal temperature, and days in milk at the time of sample collection, and antimicrobial treatment in the last 14 days with either ceftiofur or tetracycline drug. Farm was controlled as a random effect in the model. The Akaike information criterion (AIC) was used for model selection and to ensure a more parsimonious model was selected. Clinical presentation group was forced into all models regardless of the *P*-value. Confounding effects were evaluated by examining the effect of the removing variables on the coefficients of the remaining variables. A variable was considered a confounder if the coefficient of a significant variable in the model changed ≥ 20% after removal from the model.

Mixed-effect multinomial logistic regressions were used for the analysis of binomial data for AMR categorization of an isolate for each AMDs with MIC breakpoints using the logit link function in PROC GLIMMIX; in this model the response variable was a proportion using the events/trials syntax, where the events were the number of intrauterine *E. coli* isolates at a farm with AMR to the antimicrobial drug being evaluated (events) out of the total intrauterine *E. coli* isolated from that farm (trials)^33^. More specifically, the dependent variable was the number of isolates with AMR to the antimicrobial drug being evaluated at a farm (events) out of the total intrauterine *E. coli* isolated from that farm (trials). Using this approach, the models assessed the least square means for the prevalence of AMR at the farm level. The explanatory variables offered to the model were farm-level practices, including antimicrobial drugs commonly used as first choice for treatment of metritis on the farm. Using this approach, the models assessed the association between AMR proportion at the farm level and surveyed farm practices.

A model was generated for each of the nine AMDs with MIC breakpoints to evaluate the association of farm-level prevalence of *E. coli* AMR and farm-level management practices as explanatory variables. Individual models were created for ampicillin, ceftiofur, chlortetracycline, oxytetracycline, florfenicol, gentamicin, danofloxacin, spectinomycin, and enrofloxacin. Models were built and evaluated as previously described.

### Heat maps for isolate susceptibility to antimicrobials

Heat maps representing each individual isolate and its susceptibility to 12 antimicrobials by clinical presentation group and farm were created using RStudio (Version 1.4.1106) (R Foundation for Statistical Computing, Vienna, Austria) using the heatmap.2 function. Of the eighteen total drugs tested, five drugs were not included (tiamulin, sulfadimethoxine, trimethoprim-sulfamethoxazole, tylosin, and clindamycin) because these drugs had either fewer than two antimicrobial concentrations tested, or more than 98% of isolates had the same MIC value. The percentile scale for susceptibility to antimicrobials was generated after categorizing MIC dilution ranges available for each antimicrobial in ascending order, representing the percent decrease in susceptibility for the evaluated range. As an example, for oxytetracycline, five antimicrobial dilution concentrations were available in the MIC plate (0.5, 1, 2, 4, and 8 μg/mL), generating a percentile decrease in susceptibility with increments of 25%, assigned a percent category of decreased susceptibility 0%, 25%, 50%, 75%, and 100%.

## Supplementary Information

Supplementary Information.

## Data Availability

The datasets used and/or analyzed during the current study are available from the corresponding author upon reasonable request.
