# Supplementary material for: Evaluation of antimicrobial resistance and risk factors for recovery of intrauterine Escherichia coli from cows with metritis on California commercial dairy farms
Source: Sci Rep. 2022 Aug 17;12:13937. doi: 10.1038/s41598-022-18347-w (PMC9386028; doi:10.1038/s41598-022-18347-w)
Supplement: Supplementary file 1 — Supplementary Information. [file 41598_2022_18347_MOESM1_ESM.docx]

**Supplemental Figure 1.** Visual examples of oxytetracycline susceptibility determination based on growth patterns in minimum inhibitory concentration (MIC) microtiter plates.


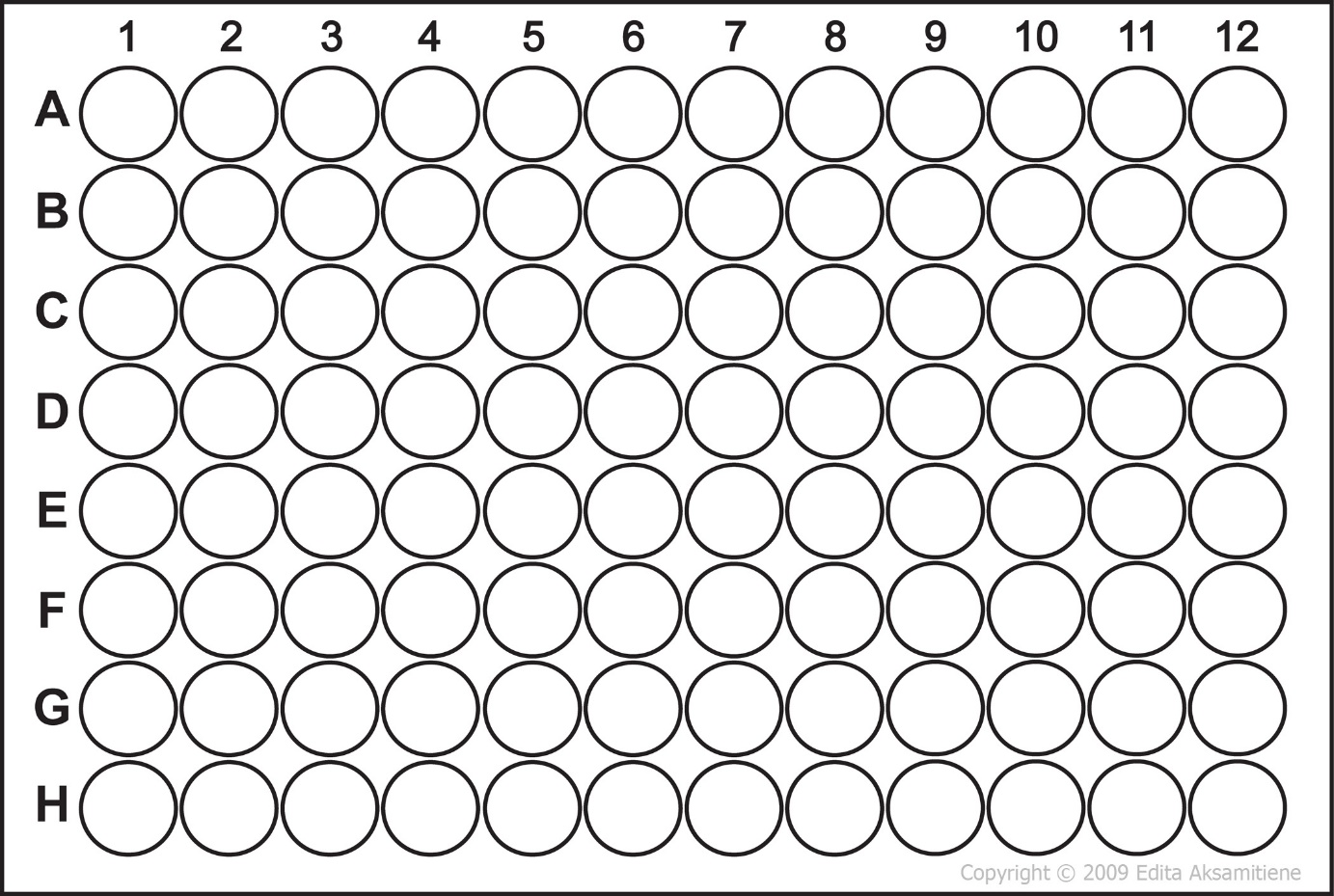


0.5 1 2 4 8

Antibiotic dilutions in µg/mL

A. MIC = 2 µg/mL; Classified as susceptible

B. MIC = 4 µg/mL; Classified as intermediate or non-susceptible

C. MIC = 8 µg/mL; Classified as resistant

D. MIC > 8 µg/mL; Classified as “Growth in all dilutions” or GAD

**Supplemental Table 1.**  Outcome for univariate analysis evaluating individual animal factors associated with the odds of isolating intrauterine *E. coli* from cows on commercial dairy farms in California.

| Variable | χ^2^ | *P* value^1^ |
| --- | --- | --- |
| Clinical Group^2^ | 10.32 | **0.005** |
| Farm^3^ | 55.98 | **0.002** |
| DIM^4^ | 12.8 | **0.003** |
| Rectal Temp^5^ | 1.72 | 0.18 |
| Lactation^6^ | 2.62 | 0.27 |

1. *P-*value for univariate analysis evaluating the association between the referred variable and the odds of isolating intrauterine *E. coli*.

2. Clinical presentation group of cows when intrauterine samples were collected. (MET) metritis discharge defined as a watery, red or brown colored, and fetid vaginal discharge; (PUS) purulent discharge defined as cows with a non-fetid purulent or mucopurulent vaginal discharge; and (CTL) control, healthy discharge defined as cows with either no vaginal discharge, clear mucus, or clear lochia.

3. Farm where cows were sampled (n = 25)

4. Days in milk

5. Rectal temperature of cows when intrauterine samples were collected.

6. Categorical variable with three levels: first, second, third, or greater lactations.

**Supplemental Table 2.** Most frequently observed resistance profiles for intrauterine *E. coli* isolates

| Drug Resistance Profile^1^ | Count  (n = 162) | Percent of Isolates^2^ |
| --- | --- | --- |
| Amp | 97 | 59.9 |
| AmpChtetOxtet | 30 | 18.5 |
| AmpOxtet | 8 | 4.9 |
| AmpChtetFlorOxtet | 5 | 3.1 |
| AmpXnlChtetFlorOxtet | 5 | 3.1 |
| AmpXnlChtetOxtet | 4 | 2.5 |
| AmpDano | 3 | 1.9 |
| AmpFlor | 3 | 1.9 |
| AmpChtet | 2 | 1.2 |
| AmpChtetOxtetDano | 2 | 1.2 |
| AmpXnlChtetOxtetDano | 1 | 0.6 |
| AmpXnlFlor | 1 | 0.6 |
| AmpXnlGenEnroDano | 1 | 0.6 |
| AmpXnlGenEnroDano | 1 | 0.6 |

1. Drugs for which isolate displayed phenotypic resistance. Amp: ampicillin, Xnl: ceftiofur, Chtet: chlortetracycline, Flor: florfenicol, Genta: gentamicin, Oxytet: oxytetracycline, and Enro: enrofloxacin.

2. Proportion of isolates displaying resistance profile out of 162 *E. coli* isolates tested for antimicrobial resistance

**Supplemental Table 3.** Antimicrobial resistance profiles for intrauterine *E. coli* isolates selected for MIC determination

| \| Isolate Number \| Clinical Group^1^ \| Resistance Profile^2^ \| \| --- \| --- \| --- \| \| 1 \| CTL \| Amp \| \| 2 \| MET \| Amp \| \| 3 \| MET \| Amp \| \| 4 \| MET \| Amp \| \| 5 \| CTL \| Amp \| \| 6 \| CTL \| AmpChtet \| \| 7 \| PUS \| AmpDano \| \| 8 \| CTL \| AmpXnlChtetFlorOxtet \| \| 9 \| MET \| Amp \| \| 10 \| CTL \| Amp \| \| 11 \| MET \| AmpChtetOxtet \| \| 12 \| MET \| AmpOxtet \| \| 13 \| MET \| AmpChtetOxtet \| \| 14 \| MET \| Amp \| \| 15 \| PUS \| Amp \| \| 16 \| PUS \| Amp \| \| 17 \| PUS \| Amp \| \| 18 \| PUS \| Amp \| \| 19 \| CTL \| Amp \| \| 20 \| CTL \| AmpOxtet \| \| 21 \| MET \| Amp \| \| 22 \| CTL \| AmpChtetOxtet \| \| 23 \| CTL \| Amp \| \| 24 \| PUS \| AmpXnlChtetOxtet \| \| 25 \| PUS \| AmpChtetOxtet \| \| 26 \| MET \| Amp \| \| 27 \| PUS \| Amp \| \| 28 \| PUS \| Amp \| \| 29 \| CTL \| AmpXnlChtetFlorOxtet \| \| 30 \| CTL \| Amp \| \| 31 \| CTL \| Amp \| \| 32 \| MET \| Amp \| \| 33 \| CTL \| Amp \| \| 34 \| MET \| AmpOxtet \| \| 35 \| PUS \| Amp \| \| 36 \| CTL \| Amp \| \| 37 \| MET \| Amp \| \| 38 \| PUS \| AmpChtetOxtet \| \| 39 \| CTL \| Amp \| \| 40 \| CTL \| AmpChtetFlorOxtet \| \| 41 \| CTL \| Amp \| \| 42 \| PUS \| AmpFlor \| \| 43 \| CTL \| Amp \| \| 44 \| PUS \| Amp \| \| 45 \| MET \| AmpDano \| \| 46 \| PUS \| Amp \| \| 47 \| MET \| AmpXnlGenEnroDano \| \| 48 \| MET \| AmpXnlChtetOxtetDano \| \| 49 \| PUS \| Amp \| \| 50 \| MET \| AmpChtetOxtet \| \| 51 \| MET \| Amp \| \| 52 \| CTL \| Amp \| \| 53 \| MET \| Amp \| \| 54 \| PUS \| Amp \| \| 55 \| PUS \| Amp \| \| 56 \| PUS \| Amp \| \| 57 \| CTL \| Amp \| \| 58 \| CTL \| Amp \| \| 59 \| MET \| Amp \| \| 60 \| MET \| Amp \| \| 61 \| MET \| AmpXnlChtetOxtet \| \| 62 \| PUS \| Amp \| \| 63 \| CTL \| AmpChtetOxtet \| \| 64 \| MET \| Amp \| \| 65 \| PUS \| Amp \| \| 66 \| CTL \| Amp \| \| 67 \| MET \| Amp \| \| 68 \| MET \| Amp \| \| 69 \| MET \| Amp \| \| 70 \| PUS \| Amp \| \| 71 \| CTL \| Amp \| \| 72 \| PUS \| AmpChtetOxtet \| \| 73 \| MET \| AmpChtetOxtetDano \| \| 74 \| MET \| Amp \| \| 75 \| CTL \| Amp \| \| 76 \| MET \| Amp \| \| 77 \| PUS \| Amp \| \| 78 \| PUS \| AmpOxtet \| \| 79 \| CTL \| Amp \| \| 80 \| PUS \| AmpChtetOxtet \| \| 81 \| CTL \| Amp \| \| 82 \| MET \| Amp \| \| 83 \| CTL \| AmpChtetOxtet \| \| 84 \| CTL \| Amp \| \| 85 \| PUS \| AmpChtetOxtet \| \| 86 \| PUS \| Amp \| \| 87 \| CTL \| Amp \| \| 88 \| PUS \| AmpChtetOxtet \| \| 89 \| MET \| Amp \| \| 90 \| MET \| Amp \| \| 91 \| MET \| AmpChtetOxtet \| \| 92 \| CTL \| AmpChtetOxtet \| \| 93 \| PUS \| AmpChtetOxtet \| \| 94 \| PUS \| AmpChtetOxtet \| \| 95 \| MET \| AmpChtetOxtet \| \| 96 \| CTL \| AmpChtetOxtet \| \| 97 \| MET \| AmpChtetFlorOxtet \| \| 98 \| CTL \| AmpChtetOxtet \| \| 99 \| PUS \| Amp \| \| 100 \| MET \| Amp \| \| 101 \| CTL \| Amp \| \| 102 \| PUS \| Amp \| \| 103 \| PUS \| Amp \| \| 104 \| PUS \| Amp \| \| 105 \| MET \| Amp \| \| 106 \| MET \| Amp \| \| 107 \| MET \| Amp \| \| 108 \| PUS \| Amp \| \| 109 \| PUS \| AmpXnlChtetOxtet \| \| 110 \| CTL \| Amp \| \| 111 \| CTL \| AmpChtetFlorOxtet \| \| 112 \| CTL \| AmpFlor \| \| 113 \| CTL \| AmpChtetOxtet \| \| 114 \| CTL \| AmpXnlChtetFlorOxtet \| \| 115 \| PUS \| AmpChtetFlorOxtet \| \| 116 \| PUS \| AmpXnlChtetFlorOxtet \| \| 117 \| MET \| AmpChtetOxtet \| \| 118 \| MET \| AmpChtetOxtet \| \| 119 \| CTL \| AmpFlor \| \| 120 \| CTL \| Amp \| \| 121 \| PUS \| Amp \| \| 122 \| MET \| AmpChtetOxtet \| \| 123 \| PUS \| AmpXnlChtetOxtet \| \| 124 \| CTL \| AmpChtetFlorOxtet \| \| 125 \| CTL \| AmpChtetOxtet \| \| 126 \| CTL \| AmpDano \| \| 127 \| PUS \| Amp \| \| 128 \| PUS \| Amp \| \| 129 \| PUS \| Amp \| \| 130 \| PUS \| Amp \| \| 131 \| MET \| Amp \| \| 132 \| MET \| AmpChtetOxtet \| \| 133 \| MET \| AmpOxtet \| \| 134 \| MET \| AmpChtetOxtet \| \| 135 \| MET \| Amp \| \| 136 \| CTL \| AmpChtetOxtet \| \| 137 \| CTL \| AmpChtetOxtet \| \| 138 \| CTL \| Amp \| \| 139 \| CTL \| AmpOxtet \| \| 140 \| MET \| AmpXnlFlor \| \| 141 \| PUS \| Amp \| \| 142 \| MET \| Amp \| \| 143 \| MET \| AmpChtet \| \| 144 \| MET \| Amp \| \| 145 \| PUS \| AmpChtetOxtetDano \| \| 146 \| PUS \| Amp \| \| 147 \| MET \| Amp \| \| 148 \| MET \| Amp \| \| 149 \| MET \| AmpChtetOxtet \| \| 150 \| MET \| Amp \| \| 151 \| MET \| Amp \| \| 152 \| PUS \| AmpXnlChtetFlorOxtet \| \| 153 \| CTL \| Amp \| \| 154 \| PUS \| Amp \| \| 155 \| CTL \| Amp \| \| 156 \| MET \| AmpChtetOxtet \| \| 157 \| CTL \| Amp \| \| 158 \| PUS \| Amp \| \| 159 \| CTL \| Amp \| \| 160 \| CTL \| AmpOxtet \| \| 161 \| CTL \| AmpOxtet \| \| 162 \| MET \| Amp \| |  |  |
| --- | --- | --- | --- | --- | --- | --- | --- | --- | --- | --- | --- | --- | --- | --- | --- | --- | --- | --- | --- | --- | --- | --- | --- | --- | --- | --- | --- | --- | --- | --- | --- | --- | --- | --- | --- | --- | --- | --- | --- | --- | --- | --- | --- | --- | --- | --- | --- | --- | --- | --- | --- | --- | --- | --- | --- | --- | --- | --- | --- | --- | --- | --- | --- | --- | --- | --- | --- | --- | --- | --- | --- | --- | --- | --- | --- | --- | --- | --- | --- | --- | --- | --- | --- | --- | --- | --- | --- | --- | --- | --- | --- | --- | --- | --- | --- | --- | --- | --- | --- | --- | --- | --- | --- | --- | --- | --- | --- | --- | --- | --- | --- | --- | --- | --- | --- | --- | --- | --- | --- | --- | --- | --- | --- | --- | --- | --- | --- | --- | --- | --- | --- | --- | --- | --- | --- | --- | --- | --- | --- | --- | --- | --- | --- | --- | --- | --- | --- | --- | --- | --- | --- | --- | --- | --- | --- | --- | --- | --- | --- | --- | --- | --- | --- | --- | --- | --- | --- | --- | --- | --- | --- | --- | --- | --- | --- | --- | --- | --- | --- | --- | --- | --- | --- | --- | --- | --- | --- | --- | --- | --- | --- | --- | --- | --- | --- | --- | --- | --- | --- | --- | --- | --- | --- | --- | --- | --- | --- | --- | --- | --- | --- | --- | --- | --- | --- | --- | --- | --- | --- | --- | --- | --- | --- | --- | --- | --- | --- | --- | --- | --- | --- | --- | --- | --- | --- | --- | --- | --- | --- | --- | --- | --- | --- | --- | --- | --- | --- | --- | --- | --- | --- | --- | --- | --- | --- | --- | --- | --- | --- | --- | --- | --- | --- | --- | --- | --- | --- | --- | --- | --- | --- | --- | --- | --- | --- | --- | --- | --- | --- | --- | --- | --- | --- | --- | --- | --- | --- | --- | --- | --- | --- | --- | --- | --- | --- | --- | --- | --- | --- | --- | --- | --- | --- | --- | --- | --- | --- | --- | --- | --- | --- | --- | --- | --- | --- | --- | --- | --- | --- | --- | --- | --- | --- | --- | --- | --- | --- | --- | --- | --- | --- | --- | --- | --- | --- | --- | --- | --- | --- | --- | --- | --- | --- | --- | --- | --- | --- | --- | --- | --- | --- | --- | --- | --- | --- | --- | --- | --- | --- | --- | --- | --- | --- | --- | --- | --- | --- | --- | --- | --- | --- | --- | --- | --- | --- | --- | --- | --- | --- | --- | --- | --- | --- | --- | --- | --- | --- | --- | --- | --- | --- | --- | --- | --- | --- | --- | --- | --- | --- | --- | --- | --- | --- | --- | --- | --- | --- | --- | --- | --- | --- | --- | --- | --- | --- | --- | --- | --- | --- | --- | --- | --- | --- | --- | --- | --- | --- | --- | --- | --- | --- | --- | --- | --- | --- | --- | --- | --- | --- | --- | --- | --- | --- | --- | --- | --- | --- | --- | --- | --- | --- | --- | --- | --- | --- | --- | --- | --- | --- | --- | --- | --- | --- | --- | --- | --- | --- | --- | --- | --- | --- | --- | --- | --- | --- | --- | --- | --- | --- | --- | --- | --- | --- | --- | --- | --- | --- | --- | --- | --- | --- |

1. Clinical presentation group of cows when intrauterine samples were collected. (MET) metritis discharge defined as a watery, red or brown colored, and fetid vaginal discharge; (PUS) purulent discharge defined as cows with a non-fetid purulent or mucopurulent vaginal discharge; and (CTL) control, healthy discharge defined as cows with either no vaginal discharge, clear mucus, or clear lochia.

2. Drugs for which isolate displayed phenotypic resistance. Amp: ampicillin, Xnl: ceftiofur, Chtet: chlortetracycline, Flor: florfenicol, Genta: gentamicin, Oxytet: oxytetracycline, and Enro: enrofloxacin

**Supplemental Table 4.** Outcome for Fisher’s Exact Test analysis evaluating effect of individual animal treatment with any antimicrobial 14 days prior to sampling on AMR in *E. coli* to nine drugs tested for with MIC breakpoints.

| Drug Resistance^1^ | OR^2^ | 95% CI OR^3^ | *P* value^4^ |
| --- | --- | --- | --- |
| Ampicillin | -* | - | ** |
| Ceftiofur | 3.0 | 0.73-12.3 | 0.13 |
| Chlortetracycline | 0.87 | 0.29-2.6 | 1.0 |
| Florfenicol | 1.38 | 0.28-6.7 | 0.66 |
| Gentamicin | -* | - | 0.11 |
| Oxytetracycline | 0.72 | 0.24-2.14 | 0.79 |
| Enrofloxacin | -* | - | 0.11 |
| Spectinomycin | -* | - | ** |
| Danofloxacin | 3.48 | 0.62-19.4 | 0.18 |

1. Resistance to antibiotic test against

2. Odds ratio for *E. coli* in the cows being treated with any antibiotic on antibiotic resistant to the referred drug when compared to cows not being treated with ceftiofur.

3. The 95% confidence interval of the odds ratio

4. *P-*value for univariate analysis evaluating the association between antimicrobial treatment of individual cows and increased resistance to drugs tested

*Odds ratio could not be calculated because one of more of the 2x2 cells were populated with a zero (no events in one of the treated vs antibiotic resistance combinations).

** All isolates susceptible or resistant; unable to evaluate effect of drug treatment on AMR

**Supplemental Table 5.** Outcome for Fisher’s Exact Test analysis evaluating effect of individual animal treatment with ceftiofur 14 days prior to sampling on AMR in *E. coli* to nine drugs tested for with available MIC breakpoints. Thirteen of the 162 cows from which *E. coli* was isolated were treated with ceftiofur.

| Drug^1^ | OR^2^ | 95% CI OR^3^ | *P* value^4^ |
| --- | --- | --- | --- |
| Ampicillin | -* | - | ** |
| Ceftiofur | 4.67 | 1.09-20.01 | 0.05 |
| Chlortetracycline | 0.67 | 0.18-2.56 | 0.76 |
| Florfenicol | 2.08 | 0.41-10.47 | 0.31 |
| Gentamicin | -* | - | 0.08 |
| Oxytetracycline | 0.55 | 0.15-2.12 | 0.55 |
| Enrofloxacin | -* | - | 0.08 |
| Spectinomycin | -* | - | ** |
| Danofloxacin | 5.24 | 0.91-30.15 | 0.10 |

1. Drug in which prevalence of AMR was tested for

2. Odds ratio for *E. coli* in the cows being treated with ceftiofur on antibiotic resistant to the referred drug when compared to cows not being treated with ceftiofur.

3. The 95% confidence interval of the odds ratio

4. *P-*value for univariate analysis evaluating the association between ceftiofur treatment of individual cows and increased resistance to drugs tested

*Odds ratio could not be calculated because one of more of the 2x2 cells were populated with a zero (no events in one of the treated vs antibiotic resistance combinations).

** All isolates susceptible or resistant; unable to evaluate effect of drug treatment on AMR

**Supplemental Table 6.** Outcome for Fisher’s Exact Test analysis evaluating effect of individual animal treatment with ceftiofur 14 days prior to sampling, stratified by clinical presentation groups (CTL, MET, or PUS), on AMR in *E. coli* to nine drugs tested for with available MIC breakpoints. Spectinomycin was omitted as all isolates were susceptible to the drug. Ampicillin was also omitted as all isolates were resistant to the drug. Thirteen of the 162 cows from which *E. coli* was isolated were treated with ceftiofur. One of 54 CTL cows, nine of 58 MET cows, and three of 50 PUS cows were treated with ceftiofur.

| Drug^1^ | OR^2^ | 95% CI OR^3^ | *P* value^4^ | |  |
| --- | --- | --- | --- | --- | --- |
| Ceftiofur |  |  |  | |  |
| CTL^5^ | -* | - | 1.0 | |  |
| MET | 6.7 | 0.8-55.6 | 0.11 | |  |
| PUS | 5.4 | 0.4-73.1 | 0.28 | |  |
| Chlortetracycline |  |  |  | |  |
| CTL | -* | - | 0.31 | |  |
| MET | 0.26 | 0.03-2.24 | 0.26 | |  |
| PUS | 1.18 | 0.10-14.08 | 1.0 | |  |
| Florfenicol |  |  |  | |  |
| CTL | -* | - | 0.15 | |  |
| MET | -* | - | 1.0 | |  |
| PUS | 7.33 | 0.51-105.91 | 0.23 | |  |
| Gentamicin |  |  |  | |  |
| CTL | -* | - | ** | |  |
| MET | -* | - | 0.16 | |  |
| PUS | -* | - | ** | |  |
| Oxytetracycline |  |  |  | |  |
| CTL | -* | - | 0.37 | |  |
| MET | 0.22 | 0.02-1.86 | 0.25 | |  |
| PUS | 1.07 | 0.09-12.71 | 1.0 | |  |
|  |  |  |  | |  |
| Enrofloxacin |  |  |  |  | |
| CTL | -* | - | ** |  | |
| MET | -* | - | 0.16 |  | |
| PUS | -* | - | ** |  | |
| Danofloxacin |  |  |  |  | |
| CTL | -* | - | 1.0 |  | |
| MET | 6.71 | 0.81-55.64 | 0.11 |  | |
| PUS | -* | - | 1.0 |  | |

1. Drug in which prevalence of AMR was tested for

2. Odds ratio for *E. coli* in the cows being treated with ceftiofur on antibiotic resistant to the referred drug when compared to cows not being treated with ceftiofur.

3. The 95% confidence interval of the odds ratio

4. *P-*value for Fisher’s Exact test analysis evaluating the association between ceftiofur treatment of individual cows within specific clinical presentation and increased resistance to drug tested

5. Clinical presentation group (CTL, MET, or PUS) of cows when intrauterine samples were collected. (CTL) control, healthy discharge defined as cows with either no vaginal discharge, clear mucus, or clear lochia; (MET) metritis discharge defined as a watery, red or brown colored, and fetid vaginal discharge; and (PUS) purulent discharge defined as cows with a non-fetid purulent or mucopurulent vaginal discharge.

*Odds ratio could not be calculated because one of more of the 2x2 cells were populated with a zero (no events in one of the treated vs antibiotic resistance combinations).

** All isolates susceptible or resistant; unable to evaluate effect of drug treatment on AMR

**Supplemental Table 7.** Outcome for Fisher’s Exact Test analysis evaluating effect of individual animal treatment with tetracyclines (chlortetracycline and oxytetracycline) 14 days prior to sampling on AMR in *E. coli* to nine drugs tested for with available MIC breakpoints. Four of the 162 cows from which *E. coli* was isolated were treated with tetracyclines.

| Drug^1^ | OR^2^ | 95% CI OR^3^ | | | *P* value^2^ | |
| --- | --- | --- | --- | --- | --- | --- |
| Ampicillin | -* | | - | ** | |  |
| Ceftiofur | -* | | - | 1.0 | |  |
| Chlortetracycline | 2.36 | | 0.32-17.27 | 0.59 | |  |
| Florfenicol | -* | | - | 1.0 | |  |
| Gentamicin | -* | | - | 1.0 | |  |
| Oxytetracycline | 1.98 | | 0.27-14.46 | 0.61 | |  |
| Enrofloxacin | -* | | - | 1.0 | |  |
| Spectinomycin | -* | | - | ** | |  |
| Danofloxacin | -* | | - | 1.0 | |  |

1. Drug in which prevalence of AMR was tested

2. Odds ratio for *E. coli* in the cows being treated with tetracycline on antibiotic resistant to the referred drug when compared to cows not being treated with ceftiofur.

3. The 95% confidence interval of the odds ratio

4. *P-*value for univariate analysis evaluating the association between tetracycline treatment of individual cows and increased resistance to drugs tested

*Odds ratio could not be calculated because one of more of the 2x2 cells were populated with a zero (no events in one of the treated vs antibiotic resistance combinations).

** All isolates susceptible or resistant; unable to evaluate effect of drug treatment on AMR

**Supplemental Table 8.** Outcome for univariate analysis evaluating effect of individual animal treatment with tetracyclines (chlortetracycline and oxytetracycline) 14 days prior to sampling, stratified by clinical presentation (CTL, MET, or PUS), on AMR in *E. coli* to nine drugs tested for with available MIC breakpoints. Spectinomycin omitted as all isolates were susceptible to the drug. Ampicillin also omitted as all isolates were resistant to the drug. Four of the 162 cows from which *E. coli* was isolated were treated with ceftiofur. None of 54 CTL cows, two of 58 MET cows, and two of 50 PUS cows were treated with tetracyclines. As no CTL cows received tetracycline, analysis could not be conducted for CTL cows.

| Drug^1^ | OR^2^ | 95% CI OR^3^ | *P* value^4^ |
| --- | --- | --- | --- |
| Ceftiofur |  |  |  |
| MET^5^ | -* | - | 1.0 |
| PUS | -* | - | 1.0 |
| Chlortetracycline |  |  |  |
| MET | -* | - | 0.08 |
| PUS | -* | - | 1.0 |
| Florfenicol |  |  |  |
| MET | -* | - | 1.0 |
| PUS | -* | - | 1.0 |
| Gentamicin |  |  |  |
| MET | -* | - | 1.0 |
| PUS | -* | - | ** |
| Oxytetracycline |  |  |  |
| MET | -* | - | 0.10 |
| PUS | -* | - | 1.0 |
| Enrofloxacin |  |  |  |
| MET | -* | - | 1.0 |
| PUS | -* | - | ** |
| Danofloxacin |  |  |  |
| MET | -* | - | 1.0 |
| PUS | -* | - | 1.0 |

1. Drug in which prevalence of AMR was tested for

2. Odds ratio for *E. coli* in the cows being treated with ceftiofur on antibiotic resistant to the referred drug when compared to cows not being treated with ceftiofur.

3. The 95% confidence interval of the odds ratio

4. *P-*value for Fisher’s Exact test analysis evaluating the association between ceftiofur treatment of individual cows within specific clinical presentation and increased resistance to drug tested

5. Clinical presentation group (CTL, MET, or PUS) of cows when intrauterine samples were collected. (CTL) control, healthy discharge defined as cows with either no vaginal discharge, clear mucus, or clear lochia; (MET) metritis discharge defined as a watery, red or brown colored, and fetid vaginal discharge; and (PUS) purulent discharge defined as cows with a non-fetid purulent or mucopurulent vaginal discharge.

*Odds ratio could not be calculated because one of more of the 2x2 cells were populated with a zero (no events in one of the treated vs antibiotic resistance combinations).

** All isolates susceptible or resistant; unable to evaluate effect of drug treatment on AMR

**Supplemental Table 9.** Antimicrobials included in the BOPO6F Vet Antimicrobial Susceptibility Testing Plate, dilution ranges, and breakpoints for *E. coli* isolates (µg/mL).

|  |  |  | **Breakpoints*** | | | |  | |  | |
| --- | --- | --- | --- | --- | --- | --- | --- | --- | --- | --- |
| **Antimicrobial Class** | **Antimicrobial Drug** | **Dilution Range** | **S** | **I** | | **R** | **Organism** | **Source or Comment** | |  |
| **Cephalosporins** | Ceftiofur | 0.25-8 | ≤2 | 4 | ≥8 | | *E. coli* | CLSI VET01S ED5- Mastitis in cattle | |  |
| **Pleuromutilins** | Tiamulin | 0.5-32 | - | - | - | |  | ** | |  |
| **Tetracyclines** | Chlortetracycline | 0.5-8 | ≤2 | 4 | ≥8 | | *Pasturella multocida* | CLSI VET01S ED5- Respiratory in cattle | |  |
| **Aminoglycosides** | Gentamicin | 1-16 | ≤2 | 4 | ≥8 | | *Enterobacterales* | CLSI VET01S ED5- Adult horse | |  |
| **Amphenicols** | Florfenicol | 0.25-8 | ≤2 | 4 | ≥8 | | *Pasturella multocida* | CLSI VET01S ED5- Respiratory in cattle | |  |
| **Tetracyclines** | Oxytetracycline | 0.5-8 | ≤2 | 4 | ≥8 | | *Pasturella multocida* | CLSI VET01S ED5- Respiratory in cattle | |  |
| **Penicillins** | Penicillin | 0.12-8 |  |  |  | |  |  | |  |
| **Penicillins** | Ampicillin | 0.25-16 | ≤0.03 | 0.06-0.12 | ≥0.25 | | *E. coli* | CLSI VET01S ED5- Metritis in cattle | |  |
| **Fluoroquinolones** | Danofloxacin | 0.12-1 | ≤0.25 | 0.5 | ≥1 | | *Pasturella multocida* | CLSI VET01S ED5- Respiratory in cattle | |  |
| **Sulfonamides** | Sulphadimethoxine | 256 |  |  |  | |  | ⁎⁎ | |  |
| **Aminoglycosides** | Neomycin | 4-32 | - | - | - | |  | ⁎⁎ | |  |
| **Folate pathway antagonist** | Trimethoprim / sulfamethoxazole | 2 / 38 | - | - | - | |  | ⁎⁎ | |  |
| **Aminocyclitols** | Spectinomycin | 8--64 | ≤32 | 64 | ≥128 | | *Pasturella multocida* | CLSI VET01S ED5- Respiratory in cattle | |  |
| **Macrolides** | Tylosin | 0.5-4 | - | - | - | |  | ⁎⁎ | |  |
| **Macrolides** | Tulathromycin | 1-64 | ≤16 | 32 | ≥64 | | *Pasturella multocida* | CLSI VET01S ED5- Respiratory in cattle | |  |
| **Macrolides** | Tilmicosin | 4-64 | - | - | - | |  | ⁎⁎ | |  |
| **Lincoamides** | Clindamycin | 0.25-16 | - | - | - | |  | ** | |  |
| **Fluoroquinolones** | Enrofloxacin | 0.12-2 | ≤0.25 | 0.5-1 | ≥2 | | *Pasturella multocida* | CLSI VET01S ED5- Respiratory in cattle | |  |

⁎ *“*S” susceptible, “I” intermediate, and “R” is resistant.

** Breakpoints not available for Gram negative bacteria from large animals

**Supplemental Table 10**. Treatment with any antimicrobials of the cows sampled according to clinical presentation group.

|  |  | Antimicrobial Treatments^2^ | |  | |
| --- | --- | --- | --- | --- | --- |
| Clinical Group^1^ | **Ampicillin** | **Ceftiofur** | **Tetracyclines** | | **N^3^** |
| CTL | 0 | 1 | 0 | | **54** |
| MET | 1 | 9 | 2 | | **58** |
| PUS | 0 | 3 | 2 | | **50** |

1. Clinical presentation group (CTL, MET, or PUS) of cows when intrauterine samples were collected. (CTL) control, healthy discharge defined as cows with either no vaginal discharge, clear mucus, or clear lochia; (MET) metritis discharge defined as a watery, red or brown colored, and fetid vaginal discharge; and (PUS) purulent discharge defined as cows with a non-fetid purulent or mucopurulent vaginal discharge.

2. Number of cows sampled given any antimicrobial treatments within 14 days prior to sampling

3. Total number of cows sampled belonging to corresponding clinical presentation group.
